# Coupling Between Hippocampal Parenchymal Fraction and Cortical Grey Matter Atrophy at Different Stages of Cognitive Decline

**DOI:** 10.3233/JAD-230124

**Published:** 2023-05-16

**Authors:** Yaqiong Xiao, Liangjun Liao, Kaiyu Huang, Shun Yao, Lei Gao, for the Alzheimer’s Disease Neuroimaging Initiative

**Affiliations:** a Center for Language and Brain, Shenzhen Institute of Neuroscience, Shenzhen, China; b Department of Neurosurgery, The First Affiliated Hospital, Sun Yat-sen University, Guangzhou, China; c Department of Radiology, Zhongnan Hospital of Wuhan University, Wuhan, China

**Keywords:** Alzheimer’s disease, brain atrophy, cognitive decline, cortical grey matter volume, hippocampal parenchymal fraction, hippocampus

## Abstract

**Background:**

Hippocampal atrophy is a significant brain marker of pathology in Alzheimer’s disease (AD). The hippocampal parenchymal fraction (HPF) was recently developed to better assess the hippocampal volumetric integrity, and it has been shown to be a sensitive measure of hippocampal atrophy in AD.

**Objective:**

To investigate the clinical relevance of hippocampal volumetric integrity as measured by the HPF and the coupling between the HPF and brain atrophy during AD progression.

**Methods:**

We included data from 143 cognitively normal (CN), 101 mild cognitive impairment (MCI), and 125 AD participants. We examined group differences in the HPF, associations between HPF and cognitive ability, and coupling between the HPF and cortical grey matter volume in the CN, MCI, and AD groups.

**Results:**

We observed progressive decreases in HPF from CN to MCI and from MCI to AD, and increases in the asymmetry of HPF, with the lowest asymmetry index (AI) in the CN group and the highest AI in the AD group. There was a significant association between HPF and cognitive ability across participants. The coupling between HPF and cortical regions was observed in bilateral hippocampus, parahippocampal gyrus, temporal, frontal, and occipital regions, thalamus, and amygdala in CN, MCI, and AD groups, with a greater involvement of temporal, occipital, frontal, and subcortical regions in MCI and AD patients, especially in AD patients.

**Conclusion:**

This study provides novel evidence for the neuroanatomical basis of cognitive decline and brain atrophy during AD progression, which may have important clinical implications for the prognosis of AD.

## INTRODUCTION

Alzheimer’s disease (AD) is a common progressive neurodegenerative disorder and recognized as the most prevalent cause of dementia. Cognitive decline in AD is found to be closely linked to brain atrophy in a variety of regions as revealed by studies using the magnetic resonance imaging (MRI) technique, and among these regions, hippocampus, a brain structure in the medial temporal lobe, has been proposed as a core neuroimaging biomarker of AD [1, 2]. In the past two decades, numerous studies have shown significant grey matter atrophy of the hippocampus in patients with both mild cognitive impairments (MCI) and AD [3, 4]. Further, many studies have also reported a right-greater-than-left asymmetry of hippocampal volume in normal aging, MCI, and AD [4–7]. Together, previous findings have suggested that atrophy and asymmetry of the hippocampus may be key features underlying the pathology of AD.

Though hippocampal volume is a direct and commonly used measurement of hippocampal atrophy, it per se may not be ideal for characterizing hippocampal degeneration in AD progression because the hippocampal volume is highly heterogeneous across subjects and strongly influenced by the individual’s brain size [8, 9]. To overcome this issue, in recent years, a novel measure, i.e., hippocampal parenchymal fraction (HPF), was developed to better characterize the volumetric integrity of the hippocampus. Specifically, the HPF is an estimate of the fraction of brain parenchyma in a predefined adaptive hippocampal region of interest (ROI), which captures the relative volume and change between brain parenchyma and cerebrospinal fluid (CSF) within the ROI. Lower HPF values indicate lower hippocampal volumetric integrity, i.e., higher hippocampal atrophy. Unlike traditional measurements of hippocampal volume, the HPF is computed quickly (<1 min per scan) and reliably (<2% failure rate), and can be applied to raw MRI images without any preprocessing [10].

Research has suggested that HPF is a sensitive marker of hippocampal atrophy in AD [10], MCI [11, 12], and even normal cognitive decline in aging [13]. It has been shown that the HPF can be used to classify stable MCI patients from those who are transitioning to AD [11]. Studies have reported that HPF values reduce with age [14, 15] and dementia severity, with AD patients having the lowest values, followed by MCI patients and then healthy controls [15]. In both normal aging and AD groups, there was right asymmetry in HPF [14, 15], and the HPF values were positively associated with cognitive ability in normal aging [14]. A few studies have shown HPF to be more sensitive than hippocampal volume in classifying AD patients and controls [10] and patients with first-episode psychosis [16]. The HPF has also been found to be a better predictor of future cognitive decline than hippocampal volume [13]. Despite these HPF findings, it is still understudied regarding whether and how the HPF is associated with cognitive decline in MCI and AD patients, leaving the clinical relevance of the HPF underlying the AD progression unknown.

A number of studies reported substantial reductions in grey matter volume in a variety of regions in MCI and AD patients as compared to cognitively normal (CN) adults, and these regions included bilateral hippocampus, parahippocampus, temporal lobes, superior and lateral temporal gyrus, parietal regions, anterior and posterior cingulate cortices, thalamus, entorhinal cortex, and cerebellum [17–21]. These findings suggest there is concurrent grey matter atrophy in the hippocampus and cortical regions. However, it is unknown how the hippocampal atrophy is coupled with cortical grey matter atrophy during the progression of AD.

In this study, we included a cohort of CN, MCI, and AD participants from the Alzheimer’s Disease Neuroimaging Initiative (ADNI) and calculated the HPF for each participant. First, we investigated hippocampal volumetric integrity during AD progression by measuring the HPF. We expected there would be progressive decreases in the HPF from CN to AD patients as reported previously [15]. Second, we examined whether and how hippocampal volumetric integrity would be associated with cognitive ability as measured by the Montreal Cognitive Assessment (MoCA), a popular screening tool for cognitive function impairment [22]. As associations between the HPF and cognitive ability have been reported in normal aging [14], we hypothesized there would be associations between the HPF and cognitive ability across participants. Lastly, we explored the coupling between hippocampal atrophy and cortical grey matter atrophy during AD progression. Specifically, we examined the associations between the average HPF value and cortical grey matter volume in the CN, MCI, and AD groups. We expected to observe different coupling patterns in these groups, with more regions involved in the MCI and AD groups, especially in the AD group.

## MATERIALS AND METHODS

### Participants

The data included in the present study were obtained from the Alzheimer’s Disease Neuroimaging Initiative (ADNI) database (https://adni.loni.usc.edu/). The ADNI was launched in 2003 as a public-private partnership, led by Principal Investigator Michael W. Weiner, MD. The primary goal of ADNI has been to test whether serial MRI, positron emission tomography (PET), other biological markers, and clinical and neuropsychological assessment can be combined to measure the progression of MCI and early AD. For more details about this database, please refer to the website http://www.adni-info.org and previous publications [23–25].

Specifically, we included 143 CN (mean age = 74.93±8 years, 62 M/81 F), 101 MCI (mean age = 73.03±9 years, 54 M/47 F), and 125 AD (mean age = 75.22±8 years, 71 M/54 F) participants in the analysis. We selected this cohort of participants because they had quality MRI images, and they were matched on age and gender across groups. All the participants had demographic information (i.e., age, gender), clinical, and cognitive measures including clinical dementia rating (CDR) and the Mini-Mental State Examination (MMSE). The majority of participants reported years of education (CN: *n* = 139; MCI: *n* = 100; AD: *n* = 123) and completed the MoCA (CN: *n* = 133; MCI: *n* = 90; AD: *n* = 74).

### MRI data collection

T1-weighted structural MRI brain scans of all 369 participants were used for this study. For detailed information regarding ADNI’s image acquisition protocols (which are different for multiple MRI scanner types used in ADNI), see http://adni.loni.usc.edu/methods/documents/mri-protocols/. Raw Digital Imaging and Communications in Medicine (DICOM) MRI scans were downloaded from the public ADNI site (http://www.loni.ucla.edu/ADNI), reviewed for quality, and automatically corrected for spatial distortion caused by gradient nonlinearity and B1 field inhomogeneity.

### Hippocampal parenchymal fraction (HPF) calculation

The HPF was computed using the KAIBA program of the Automatic Registration Toolbox (ART) (https://www.nitrc.org/projects/art). Details of this algorithm have been described elsewhere [10, 16]. Briefly, the raw 3D T1-weighted anatomical image of each participant was first visually inspected for artifacts and then reoriented to the standard anterior commissure (AC) and posterior commissure (PC) plane before the HPF calculation. The mid-sagittal plane (MSP) was determined and AC-PC cross-section on the MSP was automatically detected. A rigid-body transformation was applied on the MSP and AC-PC to a standard posterior-inferior-left (PIL) orientation. Using a priori training data and a template match, >100 landmarks in the vicinity of the ROI of the hippocampi were detected. With these detected landmarks, an affine transformation with 12 parameters was performed using least squares to map the detected landmarks on PIL space as near to their intended places as possible. The histogram of voxel intensities within the ROI was then automatically processed to determine the HPF as the proportion of non-cerebrospinal fluid (CSF) tissue identified in a ROI that was predicted to cover a normal hippocampus. HPF values were computed for the left and right hemispheres separately to obtain left HPF (LHPF) and right HPF (RHPF) values. As LHPF and RHPF values are highly correlated (*r* = 0.87, *p* < 0.001), we calculated the average of the overall bilateral measure of hippocampal volumetric integrity as the HPF value for each participant [15]:

(1) $$\mathrm{HPF}=\frac{\mathrm{LHPF}+\mathrm{RHPF}}{2}$$

The HPF values vary between 0 and 1, with lower HPF values indicating a greater degree of hippocampal atrophy.

We also estimated the asymmetry index (AI) of the HPF based on measurements of the LHPF and RHPF using the following formula [15]:

(2) $$\mathrm{AI}=\frac{\left|\mathrm{RHPF}-\mathrm{LHPF}\right|}{\mathrm{HPF}}\times 100\%$$

In accordance with previous studies [15, 26], the AI values reflect the magnitude of asymmetry without regard to the direction.

### VBM analysis

Prior to preprocessing, MR images were visually inspected and then realigned to standard AC-PC orientations. The reprocessing was implemented with the voxel-based morphometry (VBM) pipeline using the Computational Anatomy Toolbox (CAT 12, https://neuro-jena.github.io/cat/) for Statistical Parametric Mapping (SPM12, http://www.fil.ion.ucl.ac.uk), running in Matlab R2020a (MathWorks, Natick, MA, USA). Specifically, MRI images were segmented into grey matter (GM), white matter (WM), and CSF using the default tissue probability maps. Extracted GM maps were spatially normalized to a 1.5 mm Montreal Neurological Institute (MNI) template using the default DARTEL templates. Then, GM images were modulated with Jacobian determinants from the normalization procedure to preserve regional volumes. Finally, the modulated normalized GM maps were smoothed with an 8 mm full-width at half-maximum (FWHM) Gaussian kernel to enhance the signal-to-noise ratio. The voxel size of processed images was 1.5 mm×1.5 mm×1.5 mm. The total intracranial volume (TIV), total GM, WM, and CSF volume of each participant were calculated for further examination.

Homogeneity of GM images was checked via the “check data quality” function in CAT12. No outlier was detected in any group. All participants (*n* = 369) were included in the statistical analyses.

### Statistical analyses

#### Group differences in behavioral and clinical tests

Statistical analyses for demographics and neuropsychological data were performed with R software (version 3.6.3). Specifically, group differences among CN, MCI, and AD were conducted using one-way analysis of variance (ANOVA) for continuous variables (i.e., age, education, MMSE, MoCA) and Kruskal-Wallis ANOVA for categorical variables (i.e., CDR). The Chi-squared test was used to compare the differences in gender.

#### Group differences in HPF and AI

HPF and AI data were analyzed using linear regression models with the “lm” function in the R stats package. HPF or AI was the dependent variable, group as the independent variable, and age, quadratic age, gender, scanning protocol (coded as a dummy variable), and TIV were included as covariates of no interest. The specific models were as follows:

(3) $$\begin{array}{cc} \mathrm{HPF}= & \beta_{0}+\beta_{1}\times \mathrm{group}+\beta_{2}\times \mathrm{age}+\beta_{3}\\ & \times \mathrm{quadratic}\;\mathrm{age}+\beta_{4}\times \mathrm{gender}+\beta_{5}\\ & \times \mathrm{scanning}\;\mathrm{proctocol}+\beta_{6}\times \mathrm{TIV}+ɛ \end{array}$$

(4) $$\begin{array}{cc} \mathrm{AI}= & \beta_{0}+\beta_{1}\times \mathrm{group}+\beta_{2}\times \mathrm{age}+\beta_{3}\\ & \times \mathrm{quadratic}\;\mathrm{age}+\beta_{4}\times \mathrm{gender}+\beta_{5}\\ & \times \mathrm{scanning}\;\mathrm{proctocol}+\beta_{6}\times \mathrm{TIV}+ɛ \end{array}$$

In the regression models, TIV values were converted to z-scores, and the quadratic age were added to account for the non-linear dependence of HPF and AI. Both age and quadratic age were centered around the subjects’ median age (75.2 years) across participants.

#### Associations between HPF and cognitive ability

Next, we examined the associations between the HPF and cognitive ability as measured by the MoCA across participants. Specifically, the MoCA score was the dependent variable, and the HPF was the independent variable, with age, gender, education, and TIV as covariates of no interest. The specific model was as follows:

(5) $$\begin{array}{cc} \mathrm{MoCA}= & \beta_{0}+\beta_{1}\times \mathrm{HPF}*100+\beta_{2}\times \mathrm{age}+\beta_{3}\\ & \times \mathrm{gender}+\beta_{4}\times \mathrm{education}+\beta_{5}\\ & \times \mathrm{TIV}+ɛ. \end{array}$$

In the regression model, TIV values and age were converted as aforementioned. For the main effect of HPF, we ran *post-hoc* regression analysis for each group, separately, controlling for age, gender, education, and TIV.

#### Associations between HPF and GM volume

**Table 1:** Demographic and clinical information of CN, MCI, and AD groups

	CN (*n* = 143)		MCI (*n* = 101)		AD (*n* = 125)		*p*
	mean±sd	range	mean±sd	range	mean±sd	range
Gender (M/F)	62/81		54/47		71/54		0.072^†^
Age (y)	74.93±8.07	58.4–94.7	73.03±9.14	55.8–97.4	75.22±7.68	56–88	0.11
Education (y)^‡^	16.89±2.32	12–20	16.21±2.59	8–20	15.47±2.51	8–20	<0.001
CDR	0.01±0.06	0–0.5	0.48±0.15	0–1	0.8±0.33	0.5–2	<0.001^#^
MMSE	29.01±1.1	25–30	27.66±2.09	19–30	22.94±3.21	5–30	<0.001
MoCA^# #^	24.31±1.79	18–28	22.86±3.32	10–29	18.03±5.26	0–27	<0.001

^†^Result from the Chi-squared test. ^‡^Participants who reported years of education: 139 CN, 100 MCI, and 123 AD. ^#^Results from the Kruskal-Wallis ANOVA test. The rest p values from ANOVA tests. ^# #^Participants who had the MoCA scores: 133 CN, 90 MCI, and 74 AD. CDR, clinical dementia rating; MMSE, Mini-Mental State Examination; MoCA, Montreal Cognitive Assessment.

Further, we explored the associations between HPF and GM volume in the CN, MCI, and AD groups, separately. The whole brain voxel-wise correlation analysis was performed using the “y_Correlation_Image” function in DAPBI (a toolbox for Data Processing & Analysis for Brain Imaging; https://rfmri.org/dpabi) [27], with HPF as a covariate of interest, and age, quadratic age, gender, scanning protocol (coded as a dummy variable), and TIV as covariates of no interest. The resulting r maps were converted to Z maps, and the Gaussian Random-Field Theory (GRF) was used for multiple comparisons correction for each correlation map separately, with thresholds as follows: voxel-wise *p* = 0.0001, cluster-wise *p* < 0.05, two tailed (*Z* > 3.89, GRF corrected).

## RESULTS

### Demographic and clinical data

Table 1 demonstrates demographic information and cognitive measures for the CN, MCI, and AD groups. No significant differences were observed in age or gender across groups. Cognitive test scores showed significant differences among the CN, MCI, and AD groups (CDR, MMSE, and MoCA; *ps* < 0.001).

### Significant group differences in HPF

**Fig. 1:**
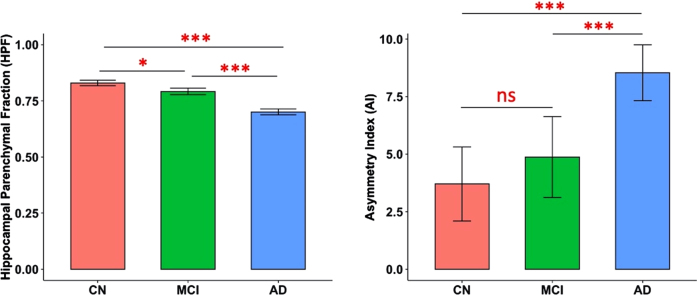
Predicted marginal means of the hippocampal parenchymal fraction (HPF, averaged across hemispheres) and asymmetry index (AI) in different groups. Both HPF and AI were found to be significantly different between CN and AD groups (*p* < 0.001) and between MCI and AD groups (*p* < 0.001); and HPF was also found significantly different between CN and MCI groups (*p* = 0.02). Error bars indicate 95% CI. ^***^p < 0.001; ^*^p < 0.05; ns, not significant.

There were significant differences in both HPF (*F*(2,363) = 114.38, *p* < 0.001) and AI (*F*(2,363) = 18.84, *p* < 0.001) across CN, MCI, and AD groups, controlling for age, quadratic age, gender, scanning protocol, and TIV. *Post-hoc* two-tailed *t*-tests demonstrated significant differences between CN and MCI groups (*t*(242) = 2.43, *p* = 0.02), between CN and AD groups (*t*(266) = 11.01, *p* < 0.001), and between MCI and AD groups (*t*(224) = 7.64, *p* < 0.001) for HPF; and significant differences between CN and AD groups (*t*(266) = 5.55, *p* < 0.001), and between MCI and AD groups (*t*(224) = 3.95, *p* < 0.001) for AI (Fig. 1).

### Significant associations between HPF and MoCA scores across groups

**Fig. 2:**
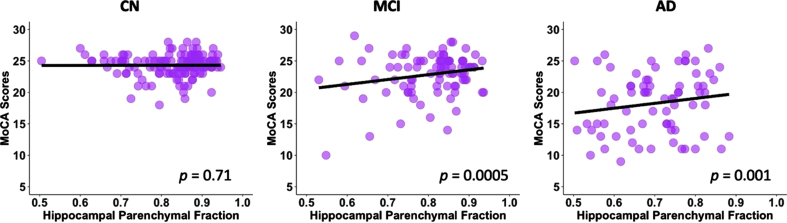
Linear regression model fitted plots between the hippocampal parenchymal fraction and MoCA scores in CN, MCI, and AD groups. Significant associations are found in the MCI (*p* = 0.0005) and AD (*p* = 0.001) groups but not in CN group (*p* = 0.71). The p values are from the *post-hoc* regression analysis.

After controlling for age, gender, education, and TIV, there was a significant association between the HPF and MoCA scores (*p* < 0.001, Table 2) across groups. For every 0.01 unit increase in HPF, the MoCA score improved by 0.23 (95% CI: [0.19,0.28]) points. In the *post-hoc* regression analysis, we found significant associations between the HPF and MoCA scores in the MCI (*p* = 0.0005) and AD (*p* = 0.001) groups, but not in the CN (*p* = 0.71) group; see Fig. 2.

### Significant associations between the HPF and cortical GM volume in CN, MCI, and AD groups

**Fig. 3:**
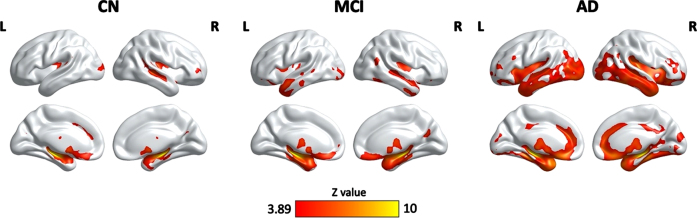
Clusters showing significant associations between the HPF and cortical grey matter volume, controlling for age, quadratic age, gender, scanning protocol, and TIV. The resulting clusters were corrected for multiple comparisons (*Z* > 3.89, GRF corrected). L, left; R, right; CN, cognitively normal; MCI, mild cognitive impairment; AD, Alzheimer’s disease.

As shown in Fig. 3, there were significant associations between HPF and GM volume in the CN, MCI, and AD groups, controlling for age, quadratic age, gender, scanning protocol, and TIV. Specifically, significant clusters included bilateral hippocampus, parahippocampal gyrus, right superior temporal cortex, middle occipital cortex, frontal regions, anterior cingulate cortex, fusiform gyrus, thalamus, caudate, and amygdala for all three groups. Significant clusters also included bilateral insula, inferior and middle template regions, temporal pole, orbitofrontal cortex, and right cuneus and calcarine for both MCI and AD groups. On top of the aforementioned regions, AD group also demonstrated significant correlation in clusters including bilateral middle cingulate cortex and cerebellum (Table 3).

**Table 2:** Estimated effects for MoCA in the linear regression model

Parameter	Estimate	Std. Error	*p*
HPF	0.23	0.023	<0.001
Age	0.16	0.028	<0.001
Gender	–0.2	0.51	0.69
Education	0.44	0.084	<0.001
TIV	0.79	0.25	0.001

## DISCUSSION

In the present study, we examined the hippocampal volumetric integrity at different stages of cognitive decline (i.e., CN, MCI, and AD) by measuring the HPF, a recently developed clinical measure of hippocampal degeneration, which reflects the relative volume and change between brain parenchyma and CSF within the predefined hippocampal region [10, 11, 13, 28]. We found progressive decreases in the HPF from CN to MCI and from MCI to AD. The asymmetry of HPF was opposite, with increasing asymmetry from CN to AD and from MCI to AD. There were positive correlations between the HPF and cognitive ability as measured by the MoCA across participants, with significant correlations in both the MCI and AD groups but not in the CN group. Further, we observed associations between the HPF and cortical grey matter volume in bilateral hippocampus, parahippocampal gyrus, superior temporal cortex, middle occipital cortex, frontal regions, anterior cingulate cortex, thalamus, caudate, and amygdala in the CN, MCI, and AD groups. The associations of HPF with cortical grey matter volume also involved bilateral insula, bilateral middle superior cortex, fusiform gyrus, inferior and middle temporal regions, superior and inferior occipital regions, orbitofrontal cortex, right cuneus, and calcarine for both the MCI and AD groups, additionally including bilateral middle cingulate cortex and cerebellum for the AD group. These findings provide evidence for the hippocampal volumetric integrity as measured by the HPF and associations of HPF with cognitive ability

**Table 3:** Clusters showing significant correlations between the HPF and GM volume in the CN, MCI, and AD groups

Region	Cluster size (voxels)	Peak MNI coordinates			Peak z value
		x	y	z
CN				
Bilateral hippocampus, parahippocampal gyrus, amygdala, fusiform gyrus, right STG, MTG	23,795	35	–26	–8	10.12
Bilateral thalamus, caudate	2,142	–8	–11	21	6.65
Left MOG	790	–21	–101	9	4.98
Left ACC	540	–12	32	30	4.8
Right IFG	415	33	53	2	4.72
MCI				
Bilateral hippocampus, parahippocampal gyrus, insula, fusiform gyrus, STG, MTG, OFC, ACC, thalamus, putamen, caudate, amygdala	52,785	–32	–23	–11	10.13
Left MTG, ITG, STG	713	–65	–39	3	4.83
Left MOG, IOG	1,154	–38	–90	6	5.39
Right precuneus, cuneus, calcarine	940	17	–66	26	5.25
Right MTG, STG	647	48	–62	18	4.85
AD				
Bilateral MTG, STG, ITG, parahippocampal gyrus, hippocampus, insula, amygdala, putamen, caudate, fusiform gyrus, MOG, IOG, OFC, ACC	116,150	36	–26	–5	10.4
Bilateral MCC	1,177	–11	–32	35	5.04
Right MFG, IFG	592	44	44	3	4.98
Bilateral cuneus, calcarine	695	9	–93	21	4.8

STG, superior temporal gyrus; MTG, middle temporal gyrus; MOG, middle occipital gyrus; ACC, anterior cingulate cortex; IFG, inferior frontal gyrus; OFC, orbitofrontal cortex; ITG, inferior temporal gyrus; IOG, inferior occipital gyrus; MFG, middle frontal gyrus; MCC, middle cingulate cortex; CN, cognitive normal; MCI, mild cognitive impairment; AD, Alzheimer’s disease.

and brain atrophy in the pathological progression of AD, and suggest that HPF may serve as a sensitive clinical marker of cognitive decline.

One of the key findings here is the association between hippocampal volumetric integrity as measured by the HPF and cortical grey matter volume in a variety of brain regions in all three groups. Notably, the strongest correlations of HPF with cortical grey matter volume were in bilateral hippocampus and parahippocampal gyrus, suggesting individuals’ HPF values are strongly related to their hippocampal volume. Moreover, there were also temporal, occipital, frontal, and subcortical regions involved in the associations of HPF with cortical grey matter volume in both MCI and AD patients, especially in AD patients. These regions are consistent with those showing greater grey matter atrophy in MCI and AD patients as compared to controls [17–21, 29, 30], suggesting a coupling between the hippocampal volumetric integrity and cortical grey matter atrophy. As such, the HPF values not only closely associate with grey matter volume in hippocampus and neighboring regions, but also reflect overall grey matter atrophy at different stages of cognitive decline. Thus, the HPF may be a sensitive marker for distinguishing patients with MCI and AD patients who have more severe cortical grey matter atrophy. Though, future studies are needed to shed light on this point.

Another key finding is that the HPF was significantly associated with MoCA scores across participants after controlling for age, gender, education, and TIV. For every 0.01 unit increase in HPF, the MoCA score improved by 0.23 points, which indicates that an increase in hippocampal volumetric integrity was correlated with an increase in cognitive ability. The association between the HPF and MoCA scores was observed in both the MCI and AD groups, but not in the CN group. In patients with MCI, lower HPF may be at a higher risk of dementia, and in patients with AD, lower HPF may be at a higher risk of more severe symptoms. While it has been previously reported that there were significant associations between the HPF and cognitive function in cognitively normal older adults [14], here we did not find correlations between the HPF and MoCA scores in the CN group. The discrepancies might be due to the small individual variability of the MoCA scores in the CN group. As the MoCA is a tool for assessing cognitive impairment [22], the individual variability of MoCA scores in CN participants (SD = 2) is relatively smaller than that in the MCI (SD = 3) and AD (SD = 5) patients. It is also possible that the associations between the HPF and cognitive ability are more salient in MCI and AD patients. In fact, research has shown the combination of clinical and cognitive measures with MRI-based hippocampal volume could increase the diagnostic confidence of AD pathology [31]. Therefore, these findings are clinically important as they suggest the evaluation of HPF provides a linkage to the neurobiological basis for cognitive decline in older adults, especially in MCI and AD patients.

We found that both the MCI and AD groups had significantly reduced hippocampal volumetric integrity than the CN group and that the AD group had significantly lower hippocampal volumetric integrity than the MCI group, controlling for age, quadratic age, gender, scanning protocol, and TIV. These results suggest that hippocampal degeneration as measured by the HPF is negatively correlated with dementia severity beyond the age, gender, scanning protocol, and total brain volume effects, which is consistent with previous studies reporting reduced hippocampal volume [18–20] and a significant decrease in the HPF with dementia severity [15].

In contrast to the hippocampal volumetric integrity, the extent of hippocampal asymmetry as measured by AI is positively correlated with dementia severity. Specifically, we found that the AD group had significantly higher hippocampal asymmetry than the MCI and CN groups. This finding is in agreement with previous studies reporting an increase in hippocampal asymmetry concurrent with disease severity controlling for age effects [6, 15]. However, the hippocampal asymmetry between the CN and MCI groups did not reach significance. According to the definition of AI, both HPF and |RHPF - LHPF| affect the AI value, and it has been shown that greater AI with an increase in disease severity could be partly due to a larger individual variability in |RHPF - LHPF| in the MCI and AD groups [15]. Thus, the lack of differences in AI between CN and MCI groups may be due to smaller individual variability in the CN group (we examined |RHPF–LHPF| between groups, but results were not reported). Another possibility is that the AI may not be as sensitive as the HPF to detect the changes in the early stages of cognitive decline.

The present study has a couple of limitations that are worth noting. First, we controlled for age, gender, education, scanning protocol, and TIV in the statistical analysis, but did not consider other factors (e.g., socioeconomic status, genetic effect, etc.) that could also affect the HPF. Second, we included cross-sectional data in this study and the findings reported here may be biased by the individual variability in different groups. Future research using the longitudinal data is needed to provide evidence for longitudinal changes in the HPF and the associations between HPF changes and changes in cognitive ability and brain atrophy during AD progression.

### Conclusion

In sum, in this study, we examined the hippocampal volumetric integrity using a recently developed measure, i.e., HPF, and investigated the associations of the HPF with cognitive ability and brain atrophy in the CN, MCI, and AD groups. Consistent with previous studies [6, 15], we found significant decreases in the HPF from CN to MCI and from MCI to AD, and significant increases in AI from CN to AD and from MCI to AD. There were positive correlations between the HPF and MoCA scores across participants, and the significant correlations were shown in both the MCI and AD groups but not in the CN group. Interestingly, we observed significant coupling between the HPF and cortical regions that show atrophy in normal aging, MCI, and AD groups, with stronger coupling (i.e., more cortical regions) in the MCI and AD groups, especially in the AD group. These findings confirm the HPF as an important clinical marker for hippocampal atrophy, and provide further evidence for the neuroanatomical basis of cognitive decline and brain atrophy during the progression of AD, which may have important clinical implications for the prognosis of AD.

## Data Availability

Data used in the present study were obtained from the Alzheimer’s Disease Neuroimaging Initiative (ADNI) database (http://adni.loni.usc.edu). The ADNI was launched in 2003 as a public-private partnership led by Principal Investigator Michael W. Weiner, MD. The primary goal of the ADNI is to test whether magnetic resonance imaging (MRI), positron emission tomography (PET), other biological markers, and clinical and neuropsychological assessment can be combined to measure the progression of mild cognitive impairment (MCI) and early Alzheimer’s disease (AD). For more information, please visit http://www.adni-info.org. The tidy data used in the current study are available at https://github.com/Yaqiongxiao/HPF.effects.AD.

## CODE AVAILABILITY

R code for implementing all the analyses can be found at https://github.com/Yaqiongxiao/HPF.effects.AD.
